# Decompressive Craniectomy in Severe Traumatic Brain Injury: The Intensivist’s Point of View

**DOI:** 10.3390/diseases11010022

**Published:** 2023-01-30

**Authors:** Matteo Vitali, Stefano Marasco, Tatsiana Romenskaya, Angela Elia, Yaroslava Longhitano, Christian Zanza, Ludovico Abenavoli, Emidio Scarpellini, Alessandro Bertuccio, Andrea Barbanera

**Affiliations:** 1 Department of Neurosurgery, AON SS. Antonio e Biagio e Cesare Arrigo H, 15121 Alessandria, Italy; 2 Department of Neurosurgery, IRCCS S. Matteo, Pavia University, 27100 Pavia, Italy; 3 Department of Anesthesia and Critical Care Medicine, St. Antonio and Biagio and Cesare Arrigo Hospital, 15121 Alessandria, Italy; 4 Foundation "Ospedale Alba-Bra” - Department of Emergency Medicine, Anesthesia and Critical Care Medicine, Michele and Pietro Ferrero Hospital, 12060 Verduno, Italy; 5 Department of Health Sciences, University “Magna Græcia”, 88100 Catanzaro, Italy; 6 Hepatology Outpatient Clinic and Internal Medicine Unit, “Madonna del Soccorso” General Hospital, 63074 San Benedetto del Tronto, Italy; 7 T.A.R.G.I.D., Gasthuisberg University Hospital, KU Leuven, Herestraat 49, 3000 Leuven, Belgium

**Keywords:** decompressive craniectomy, traumatic brain injury, intracranial hypertension, acute subdural hematoma, cranioplasty, cerebral herniation, neuro-critical care, neuro-anesthesiology

## Abstract

Introduction: Traumatic brain injury (TBI) represents a severe pathology with important social and economic concerns, decompressive craniectomy (DC) represents a life-saving surgical option to treat elevated intracranial hypertension (ICP). The rationale underlying DC is to remove part of the cranial bones and open the dura mater to create space, avoiding secondary parenchymal damage and brain herniations. The scope of this narrative review is to summarize the most relevant literature and to discuss main issues about indication, timing, surgical procedure, outcome, and complications in adult patients involved in severe traumatic brain injury, underwent to the DC. The literature research is made with Medical Subject Headings (MeSH) terms on PubMed/MEDLINE from 2003 to 2022 and we reviewed the most recent and relevant articles using the following keywords alone or matched with each other: decompressive craniectomy; traumatic brain injury; intracranial hypertension; acute subdural hematoma; cranioplasty; cerebral herniation, neuro-critical care, neuro-anesthesiology. The pathogenesis of TBI involves both primary injuries that correlate directly to the external impact of the brain and skull, and secondary injuries due to molecular, chemical, and inflammatory cascade inducing further cerebral damage. The DC can be classified into primary, defined as bone flap removing without its replacement for the treatment of intracerebral mass, and secondary, which indicates for the treatment of elevated intracranial pressure (ICP), refractory to intensive medical management. Briefly, the increased brain compliance following bone removal reflects on CBF and autoregulation inducing an alteration in CSF dynamics and so, eventual complications. The risk of complications is estimated around 40%. The main cause of mortality in DC patients is due to brain swelling. In traumatic brain injury, primary or secondary decompressive craniectomy is a life-saving surgery, and the right indication should be mandatory in multidisciplinary medical–surgical consultation.

## 1. Introduction

Decompressive craniectomy (DC) is a surgical technique developed over the centuries, and Kocher described it in detail more than a century ago. The DC has still today an essential role in the neurosurgical practice and represents a life-saving surgical option to treat elevated intracranial hypertension (ICP) [1]. The underlying rationale is to remove part of the cranial bones (“bone flap”), opening the dura mater to create space to avoid secondary parenchymal damage and brain herniations, reducing ischemic complications and improving cerebral blood flow, perfusion, and compliance.

The main indications for DC are Traumatic Brain Injury (TBI), Middle Cerebral Artery (MCA) infarction, and Acute Subdural Hematoma (ASDH); additionally, other common several pathologies (such as acute encephalitis, cerebral toxoplasmosis, subdural empyema) causing intractable elevated ICP are suitable for DC [1,2,3,4,5,6].

Primary DC is defined as bone flap removing without its replacement for the treatment of intracerebral mass, while secondary DC is indicated for the treatment of elevated intracranial pressure (ICP), refractory to intensive medical management.

The aim of this narrative review is to summarize the most relevant literature and to discuss the main issues about indication, timing, surgical procedure, outcome, and complications in adult patients involved in severe traumatic brain injury who underwent DC.

## 2. Methods

The literature search was performed on the PubMed/MEDLINE following a timeline from 2003 to 2022 with Medical Subject Headings (MeSH) terms alone or combined with each other: decompressive craniectomy; traumatic brain injury; intracranial hypertension; acute subdural hematoma; cranioplasty; cerebral herniation; neuro-critical care; and neuro-anesthesiology were the MeSH terms researched, alone or.

Our research focused on the most recent and relevant articles, and reviewed overall data about indications, timing, surgical technique, outcomes, and complications of DC and cranioplasty, excluding pediatric cases. No restrictions have been placed on the language and country of origin of the articles.

**Figure 1:**
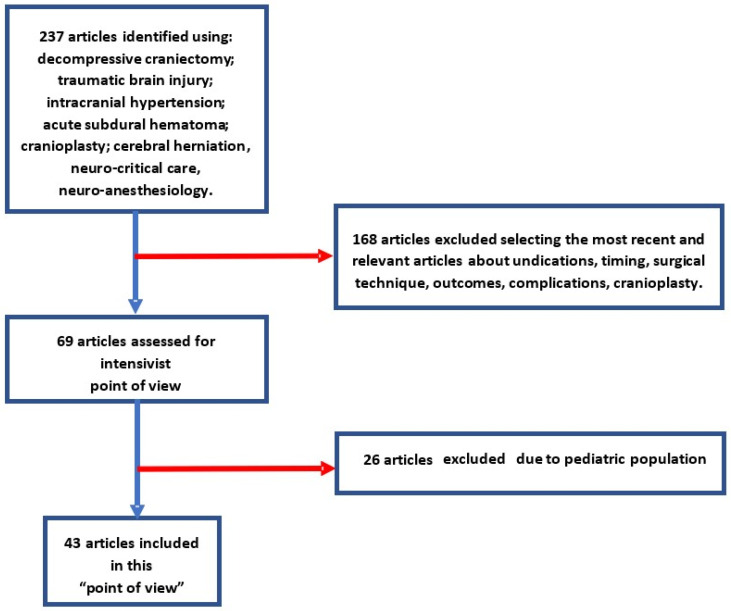
Flowchart used for the searching literature.

After careful selection, we considered about 43 articles, including two meta-analytic studies and two systematic reviews, and an International Consensus Statement (see Figure 1).

## 3. Discussion

### 3.1. Traumatic Brain Injury (TBI)

Traumatic brain injury (TBI) represents a severe pathology due to a violent blow or jolt to the head with altered state of consciousness associated with temporary or permanent neurological deficits representing social and economic concerns. TBI is one of the first causes of death and long-term disability and in Europe, the reported incidence of TBI ranges between 83.3 and 849 per 100,000 population per year [7]. The mortality rate has a wide range, accounting for from 9 to 28.10 per 100,000 population per year [7]. The highest incidence of TBI is reported in the male population aged < 44 years old, and it is related mainly to car crashes, falls, violence, sports-related, and home or work accidents [7].

### 3.2. Classification

On the basis of Glasgow Coma Scale (GCS) and the injury mechanism, TBI is classified as mild, moderate, and severe. Mild occurs in cases of alteration of alertness without neurological deficit (GCS 13–15); Moderate, usually involving patients with alteration of the consciousness with or without neurological deficits (GCS 9–12); Severe is defined in cases of a comatose or unresponsive status (GCS 3–8).

### 3.3. Pathophysiology

The pathogenesis can be primary when it correlated directly to the external impact of the brain and skull; secondary is when molecular, chemical, and inflammatory cascade inducing further cerebral damage. The primary injuries include Epidural Hematoma, Acute Subdural Hemorrhage, Subarachnoid Hemorrhage, Diffuse Axonal Injury, and Skull Fractures, with possible involvement of cranial nerves [8].

The secondary injuries are of greatest interest, because the metabolic cascade that begins after the head trauma leads to many biochemical cerebral changes, and it is very important to know to choose an appropriate therapy.

In the absence of primary lesions and/or obstructive hydrocephalus, the main cause of brain swelling, and intracranial hypertension is the cerebral edema, which is divided into two types: vasogenic and cytotoxic: although both are associated with increased ICP, the latter is more associated with severe TBI.

According to the Monro–Kellie role, the ICP is determined by the sum of blood volume, cerebral parenchymal, and Cerebrospinal Fluid (CSF). In cases of increased ICP, the self-regulation system and brain compliance maintain intracranial pressure level within physiological limit allowing an adequate cerebral perfusion pressure. In severe TBI, the loss of the cerebral compliance causes intracranial hypertension syndrome and brain herniations [9].

Vasogenic edema consists in the accumulation of water within the interstitial space, due to an alteration of the blood–brain barrier. On the other hand, cytotoxic edema involves the accumulation of water in intracellular space. The “primum movens” of this phenomenon is the alteration of the transport of ions through the cell membrane. Other contributing factors are mitochondrial dysfunction, with production of reactive nitrogen (RNS) and oxygen (ROS) species, and exotoxicity due to hyperproduction of excitatory amino acids such as glutamate [8,10].

Considering its pathogenesis and evolution, treatment of TBI can be equally complex and expensive. Mild and moderate TBI usually required clinical/radiological monitoring and medical treatment. Conversely, severe TBI is a main indication for an aggressive and fast surgical treatment as DC [6,7].

### 3.4. Decompressive Craniectomy (DC)

A meticulous historical reconstruction, based on archaeological finds, theses, and treatises, was reported by Rossini and colleagues in 2019 [11]. The first historical evidence of skull trepanation dates to about 10,000 BC, at the beginning of the Neolithic but officially the first DC FOR severe traumatic brain injury was proposed by Kocher in 1901 [11].

Because of the extreme pathological variability in the severe TBI, DC plays different roles in its management, and several Italian and international consensus conferences have been organized aiming to define the proper indications for DC [12,13,14]. Ultimately, the definition of primary and secondary DC has been suggested to discriminate between an emergency (primary DC) or an ultimate (secondary DC) surgical treatment line (see Table 1).

### 3.5. Primary DC

Indications for primary DC include all cases of intracranial lesions causing a mass effect with an eventual evolution through altered ICP and brain herniation postoperatively. While isolated epidural hematoma (EDH) and intraparenchymal contusion or hematoma represent lesions at low–medium risk, acute subdural hematoma (ASDH) is a pathological condition with high risk for developing intracranial hypertension [15]. Therefore, the main indication for a primary DC is ASDH in severe TBI.

ASDH is a relatively common finding in patients with severe TBI (about one third of cases) and in most of cases, an emergency evacuation is required. The outcome in these patients is very poor with a mortality rate ranging between 40 and 60% and a good functional recovery rate ranging between 19 and 45% [14]. In 2012, Li et al. retrospectively evaluated the outcome in 91 patients with ASDH and randomized in craniotomy versus primary DC group [16]. Results showed not significant differences between CR and DC in terms of mortality (32% vs. 38%, respectively) (*p* = 0.65). The unfavorable outcome, defined as a dead, vegetative status or severe disability, resulted less in the CR group (55%) than the DC group (58%), despite a statistically significant difference being recorded (*p* = 0.83) [16].

Similarly, Shibahashi et al. studied the in-hospital mortality and length of hospital stay in 1788 patients with ASDH who underwent CR versus primary DC. The analysis showed not a significant difference in mortality rate between the 2 groups (41.6% for the CR group vs. 39.1% for DC). Conversely, the hospital stay was significantly longer in the DC group (*p* = 0.005). Interestingly, the subgroup analyses founded a strong relation between the outcome and DC. In detail, patients with a Glasgow Coma Scale score < 9, involved in high-energy traumatic events and with a survival probability < 64%, were candidates suitable for DC [17]. In 2019, a consensus statement about the role of primary DC in severe TBI was published aiming to define the most appropriate selection criteria [13]. Results suggested considering primary DC, after the evacuation of ASDH, when intraoperatively, the brain results in bulging beyond the inner table of the skull. Conversely, the bone flap should be replaced when the brain is relaxed, and the pre-operative evaluation is not suspected of a condition with high risk of progressive brain swelling. In detail, predictive factors in favor of craniotomy are the absence of severe mass lesion, low-energy trauma, and elderly patients. Interestingly, a multicenter randomized trial aiming to evaluate the role of primary DC in patient with ASDH is currently ongoing (RESCUE-ASDH trial) [15].

### 3.6. Secondary DC

In TBI, secondary DC plays a role in the treatment of brain edema and the resultant elevated intracranial pressure (ICP) not responding to first-tier interventions. Two main multicentric randomized trials studied the role of DC in TBI: DECRA (ISRCTN61037228) [18] and RESCUEicp (ISRCTN66202560) [19]. Both studies investigated the relation between timing and outcomes in patients that underwent a secondary DC for the treatment of refractory ICP. However, the RESCUEicp trial enlarged the indication for DC to older patients and with a higher ICP threshold and a longer clinical onset. In detail, DECRA focused on an early bifronto-temporal DC for refractory ICP in 155 TBI patients [18]. Indication for an early treatment was a recording ICP above 20 mmHg for more than 15 min in a 1 h-period within the first 72 h despite the optimization of medical treatment. The short-term outcome resulted in favor of a DC group, with a better ICP control and a shorter Intensive Care Unit stay recorded (*p* < 0.001) [18]. However, these data were not confirmed at the 12-months outcome [20]. Mortality rate results were similar between the two groups. Conversely, vegetative status or a severe disability occurred more frequently in the surgery group than in the conservative group (70% vs. 51%; *p* = 0.02). At 12 months, 26% of patients in the standard care group experienced a better neurological outcome than the DC group (14%) (OR 0.33; 95% CI: 0.12–0.91; *p*  =  0.03). However, it is important to point out that although the two groups were well matched for most variants, in the DC group, there was a high proportion of patients with bilateral unreactive pupils compared to the conservative group (27% vs. 12%; *p* = 0.04) [20].

Similarly, RESCUE icp focused on late DC for refractory ICP in patients [19]. In this study, indication for a late DC was a recorded ICP above 25 mmHg in a 1-h to 12-h period within the first 10 days, despite the optimization of medical treatment. In this study, surgical treatment consisted of the fronto-temporo-parietal DC (or hemicraniectomy) or the bifrontal DC. The trial evaluated the mortality rate and the functional outcome, using the GOS-E score, at 6 (primary outcome), 12, and 24 months (secondary outcome). At primary outcome, results proved that DC guarantees a lower mortality rate (26.9% vs. 48.9% in the medical group) despite a higher rate of vegetative state and severe disability than medical management. Conversely a good functional outcome was similar in both groups (42.8% vs. 34.6%, *p* = 0.12). At 12 months follow-up, a favorable outcome occurred in 45.4% of the DC group compared with 32.4% of the conservative group (*p* = 0.12) [19]. The knowledge of probabilistic mortality and morbidity for each case and the awareness of a high rate of severe disability or vegetative state is essential in the management of severe TBI. Indeed, before surgery, the discussion with the patient’s family is crucial. The surgeon must widely explain and be sure that the patient’s family understand the long-term post-operative recovery and follow-up, and the probable persistent severe disability despite so aggressive a surgery. The aim of the discussion is to define if a DC to preserve the patient’s life is preferred despite a low quality of life.

#### 3.6.1. Surgical Techniques

DC is an urgent surgical procedure that requires both a quick operation time and adequate decompression to guarantee a reduced mortality and morbidity. Different surgical procedures have been described and classified as infratentorial or supratentorial DC.

Infratentorial or suboccipital DC is unusually employed in the treatment of severe TBI due to the rare involvement of posterior fossa in severe TBI. Conversely, it is largely indicated in cases of ischemic or hemorrhagic posterior fossa stroke [11].

Supratentorial DC is further divided into unilateral or bilateral approaches. Each technique has its own indications. In all cases, the main issue remains the extent of decompression to avoid additional brain herniation or venous infarction along the craniectomy borders and so, additional brain swelling; below is a short overview of the two most common technique (bifrontal DC and fronto-parieto-temporal DC) and the latest technique proposed.

#### 3.6.2. Bifrontal DC

Bilateral DC includes bifrontal craniectomy and bilateral frontotemporal craniectomy, aiming to decompress both hemispheres in cases of diffuse brain edema without localized lesions.

The primary indication for bifrontal DC is severe TBI with frontal contusions and diffuse brain edema. In this surgical approach, the patient is in a supine position, without head rotation. A frontal curve incision is performed anterior to the tragus on each side and extended 2 to 3 cm posterior to the coronal suture. Great care should be taken to preserve bilaterally the superficial temporal arteries (STA), being the main vascularization feeders. A musculocutaneous flap is performed and reflected forward over the orbital rim. Several burr-holes are bilaterally performed in keyhole areas, squamous parts of the temporal bones and 1 cm apart from the midline on each side, aiming to facilitate the dissection of the superior sagittal sinus (SSS). Therefore, a wide bifrontal craniectomy is completed. Durotomy is so performed, and the goal is to divide the anterior portion of SSS and underlying falx to guarantee brain expansion and to avoid herniation against a tight dural edge.

Despite this technique having been largely used in the past, the most recent guidelines do not recommend its employment [12]. Indeed, DECRA confirmed that, although effective in controlling ICP, bilateral DC does not improve long-term outcomes [18].

#### 3.6.3. Fronto-Parieto-Temporal DC or Hemicraniectomy

Unilateral DC is the most common technique and consists in a fronto-temporo-parietal craniectomy.

The patient is supine with the head turned to the contralateral side. A large cutaneous question mark-shaped incision is made. Again, great caref to preserve STA is essential to avoid ischemic complication of the flap. After dissection and reflection of the musculocutaneous flap, a fronto-parieto-temporal craniectomy is performed. As a general rule, unilateral DC should not be smaller than 12 × 15 or 15 cm in diameter and extended toward the floor of the temporal fossa to provide adequate decompression [21,22]. Indeed, small decompression could be inadequate and may cause further brain damage by compression of the brain cortex and cortical veins that so enhance brain herniation.

Additionally, dura opening with dural expansion is recommended to guarantee a more effective decompression in terms of reduced ICP and increased cerebral tissue oxygenation [23,24].

After decompression, the removed bone flap must be appropriately preserved until the subsequent cranioplasty that should require several months. Mainly, two different methods have been proposed. The first consists in the position of the bone flap in the patient’s abdominal wall in a subcutaneous fashion. Considering the added risk of this procedure, surgeons usually prefer the second method. It consists in storage in a sub-zero degrees (−20 °C)-temperature freezer by authorities named Bone Banks.

#### 3.6.4. Novel Surgical Techniques

A novel DC technique was proposed by Feng and colleagues [25]. The main difference from traditional hemicraniectomy concerns the skin incision, which is performed starting about 1 cm above the ipsilateral mastoid process and 3–4 cm posterior to the pinna. The skin incision continues anteriorly, approaching about 1–2 cm away from the midline to the hairline anteriorly. This technique allows one to perform a sufficiently large hemicraniectomy while being careful during the craniotomy in the lower and rear for the presence of mastoid air cells and venous sinuses. Additionally, with the retraction of the skin flap, the external auditory canal could be breached if care is not taken. The main advantages over the traditional technique are the easier preservation of the vascular territories of the superficial temporal artery (STA), ideal for patients suffering from large skin contusions, diabetes mellitus, or immunosuppression [25]. In addition, Veldelman et al. report a lower risk of infection performing the skin incision posteriorly to the external acoustic meatus compared to anteriorly [26].

### 3.7. Complications

After DC, pathophysiological alterations in ICP, CSF circulation, and CBF could induce complications. Briefly, the increased brain compliance following bone removal reflects on CBF and autoregulation inducing and alteration in CSF dynamics and so, eventual complications. The risk of complications is estimated at about 40%. The main cause of mortality in DC patients is due to brain swelling. Complications can be divided into two main subgroups, those directly related to DC (acute) and those related to cranioplasty (late) (see Table 2).

### 3.8. Acute Complications

Acute complications can be further divided into ultra-early, early, and delayed events.

Ultra-early complications include peri-operative events such as blossoming of contusion, epidural hematoma, external cerebral herniation, intracranial infection, epilepsy, CSF leakage, and wound problems. Blossoming of contusion is due to the development of a new expanded contusion during bone decompression causing malignant swelling or elevation of ICP [11]. During DC, the bone removal causes alteration on intracranial pressures and an increase in the hydrostatic pressure gradient, resulting in transcapillary leakage and brain edema. These alterations can cause external cerebral herniation [27].

Subdural effusions or hygromas, evolution of contralateral mass lesions, paradoxal herniation, and infection are early complications that can be observed in the first months. Conversely, delayed complications arise after 30 days from surgery and include syndrome of the sinking skin flap (SSFS) or trephined syndrome and hydrocephalus. Pathophysiology of delayed complications is mainly related to CSF dynamics derangements and venous flow impairments caused by the atmospheric pressure on intracranial cavity resulting in compliance alterations. Trephined syndrome was first described by Grant and Norcross in 1939 [28]. This syndrome included non-specific cognitive and emotional symptoms such as dizziness, fatigue, discomfort in the site of the defect, apprehension and insecurity, depression, and intolerance to vibration. In the 1970s, Yamaura and Makino used the term “syndrome of the sinking skin flap” (SSSF) to describe the development of focal neurological deficits in patients who underwent DC. The pathogenesis of the SSFS was linked to the role of atmospheric pressure on the brain inducing pathological alterations and deformations resulting in the sinking skin in the cranial defect [29]. Similarly, the term “motor trephine syndrome” was used to define the delayed development of a contralateral monoparesis in the same population [30]. Nowadays, the terms “ST”, “SSSF”, and the “motor trephined syndrome” have been replaced by the more generic term “neurological susceptibility to a skull defect” [31].

Indeed, all these syndromes found a similar pathogenesis due to alteration in CSF flow and CBF after DC and in a delayed timing before cranioplasty. Therefore, all these syndromes usually improve after cranioplasty.

### 3.9. Late Complication

Late complications are mainly related to the cranioplasty surgery [32,33]. They include bone resorption, osteomyelitis, and hypo-vascular bone necrosis.

Resorption of the bone flap (aseptic osteonecrosis) is one of the most common late complications after cranioplasty surgery, especially in children [34]. Young age and presence of ventriculoperitoneal shunt are two risk factors that increase the odds of this severe complication [34,35,36]. Resorption of the bone flap may also lead to brain tissue injury plus cosmetic damage by forming scars or keloids. To prevent this complication, it is necessary to accurately choose either the cranioplasty technique or the synthetic materials used [37].

The incidence of site infection subsequent to cranioplasty approximates at between 2.3% and 20% [38] and is highly variable depending on the type of material used in the surgical procedure. The evidence from the literature suggests that autologous cranioplasty is more at risk of developing long-term complications, such as osteomyelitis, than hydroxyapatite cranioplasty (6.9% vs. 3.3%, respectively) [39]. Alkhaibary et al. performed a retrospective analysis and found that the most significant predictors of infection in patients requiring cranioplasty were blood glucose levels and skull defect size (*p* = 0.03 and *p* = 0.02, respectively) [40].

### 3.10. Outcome

In 2020, Celi and Saal-Zapata reported their case series including 33 patients who underwent DC aiming to identify factors affecting the mortality of surgically treated TBI. The study presented significant limitations, such as the few numbers of patients and the inclusion of different decompressive techniques. However, the in-hospital mortality was higher in patients with TBI and the midline shift > 5 mm (*p* = 0.033) or larger skull flap (*p* = 0.003) [41].

## 4. Conclusions

Decompressive craniectomy is still today a life-saving surgery that is indicated in different situations, especially in patients with TBI [42,43]. Conflicting data about acute and late complications on the impact on quality of life demonstrate the need for further randomized clinical trials. In the therapeutic choice, both primary and secondary DC, and collegial consultation between anesthesiologists, intensivists, neurosurgeons, and neuroradiologists is crucial. Last but not least, the right information for the patient’s family about the risks and benefits of the surgical procedure is an essential moment of the therapeutic process.

## Figures and Tables

**Table 1:** Classification and indications of Decompressive Craniectomy.

Decompressive Craniectomy (DC)	Indications	Pathophysiology
Primary DC	Acute subdural hematoma (ASDH) in TBI and seldom lesions at low–medium risk (such as isolated epidural hematoma (EDH) and intraparenchymal contusion or hematoma)	Intracranial lesions causing a mass effect with altered ICP and brain herniation postoperatively
Secondary DC	Treatment of brain edema and the resultant elevated intracranial pressure (ICP) refractory to first-tier interventions.	The secondary injuries consist in metabolic cascade that begins after the head trauma leading to many biochemical cerebral changes (vasogenetic edema, loss of cellular homeostasis with cellular swelling mitochondrial dysfunction, RNS, ROS)

DC—Decompressive Craniectomy, ASDH—Acute subdural hematoma, EDH—epidural hematoma, ICP—intracranial pressure, RNS—reactive nitrogen, ROS—reactive oxygen.

**Table 2:** Classification of Complications after DC.

Complications	Type of Complications	
ACUTE COMPLICATIONS (Directly related to DC)	Ultra-early	Peri-operative events, such as blossoming of contusion, epidural hematoma, external cerebral herniation, intracranial infection, epilepsy, CSF leakage, and wound problems
	Early (in the first months)	Subdural effusions or hygromas, evolution of contralateral mass lesions, paradoxal herniation, and infection
	Delayed events (after 30 days from DC)	Syndrome of the sinking skin flap (SSFS) or Trephined syndrome and hydrocephalus.
LATE COMPLICATIONS (Related to cranioplasty)	Bone resorption, osteomyelitis, and hypo-vascular bone necrosis	

DC—Decompressive Craniectomy, CSF—cerebral spinal fluid, SSFS—Syndrome of the sinking skin flap.
